# Iron deficiency in patients undergoing radiotherapy: Prevalence and clinical characteristics

**DOI:** 10.1016/j.ctro.2026.101111

**Published:** 2026-01-18

**Authors:** Sophie Schumacher, Falk Fabian, Paula Lehmann, Daniel Zips, Felix Mehrhof, Wolfram Doehner, Franziska Hausmann

**Affiliations:** a Department of Radiation Oncology, Charité University Medicine Berlin, Corporate Member of Freie Universität Berlin and Humboldt-Universität Zu Berlin, Berlin, Germany; b Berlin Institute of Health, Center for Regenerative Therapies, Charité - Universitätsmedizin Berlin, Berlin, Germany; c Deutsches Herzzentrum der Charité, Department of Cardiology (Campus Virchow-Klinikum), Charité-Universitätsmedizin Berlin, Berlin, Germany; d German Center for Cardiovascular Research (DZHK), partner site Berlin, Germany

**Keywords:** Iron deficiency, Anemia, Radio-chemotherapy, Transferrin saturation, Health-related Quality of Life (HRQoL)

## Abstract

**Purpose:**

Anemia in patients undergoing radio-chemotherapy (RCTx) is often driven by iron deficiency and can impact quality of life and therapy outcomes. This study aimed to evaluate how RCTx affects iron metabolism and identify additional influencing factors.

**Methods:**

We conducted a retrospective study on 156 patients with cancer undergoing RCTx, primarily with head and neck cancer, brain malignancies, or metastatic disease. Anemia was defined as a HB level < 13 mg/L in men and < 12 mg/L in women. Iron deficiency was defined as a transferrin saturation (TSAT) below 20 %. Further blood parameters, including iron, ferritin, and transferrin, were assessed at multiple timepoints before, during, and after RCTx.

**Results:**

Prevalence of anemia was 53.8% prior to treatment and increased up to 74.1% at the end of radiotherapy. At baseline, 44.2% of patients were iron deficient. Iron deficiency at baseline was associated with systemic treatment (16.5% of patients with neoadjuvant treatment), while low TSAT at end of treatment was associated with alcohol abuse. During the course of the RCTx patients with divergent changes of TSAT levels were identified (increase, decrease and no change of TSAT). Trajectory of TSAT levels was independent of oral iron substitution as nutritional supplement.

**Conclusion:**

Patients undergoing radiotherapy show an exceptionally high prevalence for anemia, with iron deficiency being one of the main causes. Our study suggests that oral iron substitution may not be sufficient to treat iron deficiency in patients undergoing radiotherapy, underlining the need to enhance our understanding of iron balance and how to best target deficiencies in these patients.

## Highlights

• Prevalence of anemia is high and increases during the course of treatment in patients undergoing radiotherapy.
• Prevalence of iron deficiency is high already prior to radiotherapy and associated with systemic treatment.

## Introduction

Patients with cancer often suffer from cancer- or therapy induced anemia. Symptoms include fatigue, reduced physical and cognitive functional capabilities and can severely impact health-related quality of life (HRQoL). Radiotherapy-induced anaemia (RIA) occurs in around 38.2 % and rises up to 62 % in patients treated with concomitant chemotherapy [1]. Severe anemia is generally treated with red blood cell transfusions with the potential risk of a wide range of immediate and delayed reactions such as acute hemolytic transfusion reaction (AHTR) or Transfusion-related acute lung injury (TRALI) as well as milder reactions such as circulatory overload, fever and even allergic or anaphylactic reactions. The constant shortage of compatible blood reserves must also be taken into account [2]. A potential alternative is the use of erythropoiesis-stimulating agents (ESAs) and iron substitution [3].

Reasons for anemia in patients with cancer include occult or tumor bleeding, nephrotoxicity (impairing the synthesis of erythropoietin), bone marrow insufficiency and iron or other nutrient deficiency (i.g. Vitamin B12 and folic acid) [4]. In addition, cancer-related anemia, also called anemia of chronic disease (ACD), is characterized by immune activation and chronic inflammation. It also results in hepcidin-induced reduction of intestinal iron resorption, reduced iron release from enterocytes and macrophages and reduced iron uptake [5], all resulting in iron-restricted erythropoiesis. Therefore, finding and treating the cause of anemia in patients with cancer remains challenging [6]. Plasma ferritin levels have been shown to be unsuitable for indicating iron deficiency (ID) in patients with cancer, as ferritin is an acute-phase protein and thus often chronically elevated. Thus, transferrin saturation (TSAT) as an indicator of endogenous iron mobilization and transport to utilizing tissues has been applied to obtain more accurate information on iron balance in patients with cancer related iron deficient anemia. TSAT levels are calculated based on measurements of iron and transferrin in blood plasma using the following formula: TSAT [%] = iron [µg/dl] / transferrin [mg/dl] x 70.9. Overall, insights on incidence and dynamic changes of ID in patients undergoing radio(chemo-)therapy is limited thus far. Therefore, we aimed to assess the number of patients with ID prior and after radiotherapy, the changes of blood values important for the iron homeostasis during treatment and investigate potential treatment and patient characteristics being associated with ID.

## Material and methods

### Participants and recruitment process

All patients with available blood samples before starting radiotherapy (baseline) between 01.11.2022 and 31.10.2023 at the department of radiation oncology at Charité University Medicine Berlin were included in this retrospective study. Patient clinical information including medical history, radiation treatment schedules were retrieved from the hospital information system and the treatment planning system (ARIA, Varian inc., USA). The study was approved by the institutional review board of Charité University Medicine Berlin under the number EA1/101/23.

### Blood samples

Blood samples were assessed for standard lab variables including blood cell counts for erythrocytes, leucocytes, thrombocytes, neutrophils, and lymphocytes as well as levels of hemoglobin, hematocrit, c-reactive protein (CRP), Ferritin, Iron, Transferrin, and Transferrin Saturation in the routine clinical lab of the hospital. Neutrophil-to-Lymphocyte ratio (NLR) was calculated by dividing cell count for neutrophils by cell count for lymphocytes. Platelet- to-Lymphocyte ratio (PLR) was calculated by dividing cell count for platelets by cell count for lymphocytes. Repeated blood samples were obtained prior and during the radiation therapy scheme and were allocated with regard to irradiation dates (baseline (BL), intermediated time point 1(I1), time point 2 (I2), time point 3 (I3), end of radiotherapy (EOT), and follow up (FU)). Baseline blood sampling (BL) was at day of treatment start for most patients (median = 0 days; range: −44 to + 5 days) (**Suppl. Fig. 1A**). Blood sampling for time points 1 (I1), time point 2 (I2) and time point 3 (I3) were at day 13, 24, and 33 (median). For end of treatment (EOT) and follow up (FU), blood sampling was a median of −1 days and 33 days after end of radiotherapy treatment. Maximum time point of FU was 70 days. Mean irradiation doses at baseline, I1, I2, I3, EOT and FU were 0.5, 17.1, 33.1, 46.8, 53.7 and 58.8 Gy, respectively (**Suppl. Fig. 1B**). ANOVA analysis revealed significant increase of radiation dose between baseline and all other time points (p < 0.0001). Total number of available data sets per time point were 156 (BL), 37 (I1), 34 (I2), 30 (I3), 27 (EOT), and 6 (FU), respectively. For subgroup analysis of progression of ID from baseline to EOT patients with repeated measurements for TSAT were selected. We excluded 1 patient with i.v. iron supplementation from this analysis.

### Definition of anemia and ID

Anemia was defined according to the definition of the World Health Organization (WHO) as a HB level < 13 mg/L in men and < 12 mg/L in women [7]. As previously described for patients with malignancies, ID was defined as transferrin saturation (TSAT) below 20 % [8].

### Data analysis

All variables are primarily described descriptively with suitable position and dispersion measures (e.g. mean value with standard deviation or standard error, median with 95 % confidence interval). Comparison of measurements at different time points was analyzed with a repeated measures analysis of variance (ANOVA). For analysis of repeating samples paired *t*-test was performed. For bivalent analysis of correlation between nominal scaled values Chi square tests were used. For metrically scaled variables Pearson product-moment correlations were performed. P- values equal or lower than 0.05 were considered significant. For statistical analysis, SPSS Version 29 (IBM, Armonk, USA) and Graph Pad Prism Version 10.3.0 for Windows (GraphPad Software, Boston, Massachusetts USA) were used.

## Results

### Patient and treatment characteristics

A total of 156 patients were included in this study. Patient characteristics are summarized in Table 1. Mean age of patients was 64 years at time of radiotherapy treatment (range 19–86 years) and a majority of patients were male (61.5 %). Most patients presented with a tumor of the head and neck region (46.8 %) and 66.0 % of patients received systemic therapy as well as radiation. Mean radiation dose was 53.2 Gy (range 3–75.6 Gy) with a median dose per fraction of 2.2 Gy (range 0.5–15 Gy). Physical target volume was 684.4 cm^3^ on average, while gross tumor volume was 78.7 cm^3^. On average patients were 173.2 cm high, had a weight of 75.3 kg and a mean BMI of 25.1 kg/m^2^. Weight loss occurred in 46 patients (29.5 %) with a mean weight loss prior to therapy of 8.7 kg. Performance status measured by Karnofsky Index was 80 % on average. Most patients were current smokers (38.5 %).

**Table 1:** Patient characteristics; n = 156 if not otherwisely indicated. n = number of patients, BMI = body mass index, GTV = Gross Tumor Volume, Fx = Fraction, KI = Karnofsky Index, PTV = physical target volume, RT = radiotherapy, NA = not available.

Gender n(%)		
Female	60 (38.5)	
Male	96 (61.5)	
**Age [years](mean, range)**	64 (19–86)	
**Height [cm] (mean, range)**	173.2 (147–200)	*n = 129*
**Weight [kg] (mean, range)**	75.3 (34–140)	*n = 132*
**BMI [kg/m^2^] (mean, range)**	25.1 (14–39.4)	*n = 125*
**Weight loss (n, %)**		
No weight loss	65 (41.7)	
Weight loss	46 (29.5)	
NA	45 (28.8)	
**Weight loss [kg] (mean, range)**	8.7 (2–30)	
**KI [%] (median, range)**	80 (40–100)	
NA	18	
**Tumor Entity (n, %)**		
Lung	16 (10.3)	
Head & Neck	73 (46.8)	
Rectum/Anal	4 (2.6)	
Metastastatic Disease	23 (14.7)	
Prostate/Bladder	3 (1.9)	
Female pelvis	6 (3.8)	
Brain	26 (16.7)	
Esophagus	4 (2.6)	
other	1 (0.6)	
**RT total dose [Gy] (mean, range)**	53.2 (3–75.6)	
**RT dose/Fx [Gy] (median, range)**	2.2 (0.5–15)	
**Number of fractions (median, range)**	30 (1–63)	
**PTV (mean, range)[cm^3^]**	684.4 (23.7–3600.0)	*n = 155*
**GTV (mean, range)[cm^3^]**	78.7 (1.4–846.0)	*n = 115*
**Systemic therapy (n, %)**	103 (66.0)	
Chemotherapy		
Neoadjuvant	11 (10.7)	
Concurrent	88 (85.4)	
adjuvant	1 (1.0)	
Immunotherapy		
Neoadjuvant	6 (5.8)	
Concurrent	1 (1.0)	
adjuvant	4 (3,9)	
Others	4 (3,9)	
**Nicotine (n, %)**		
Current smoker	60 (38.5)	
Former smoker	19 (12.2)	
Never smoker	24 (15.4)	
NA	53 (33.9)	
**Pack years (mean, range)**	40.7 (6–120)	*n = 63*
**Alcohol abuse (n, %)**		
Current	22 (14.1)	
Former	7 (4.5)	
never	75 (48.1)	
NA	51 (32.7)	

### Anemia and ID before and at the end of treatment

**Fig. 1:**
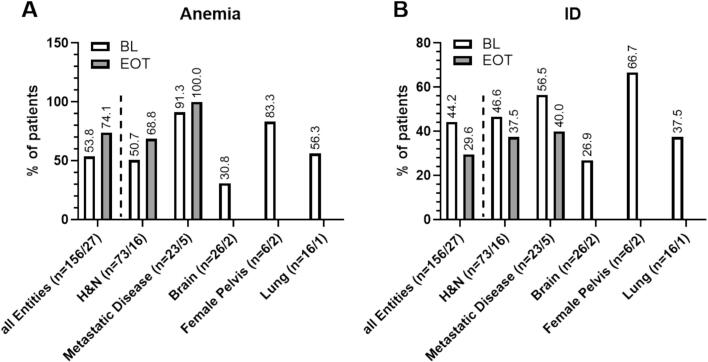
Relative prevalence of patients with anemia (A) and ID (B) at baseline (unfilled bars) and at the end of treatment (filled bars). Entities with less than n = 5 patients are not shown as individual subgroups. H&N= Patients with Head and Neck Cancer, EOT = End of Radiotherapy.

**Fig. 2:**
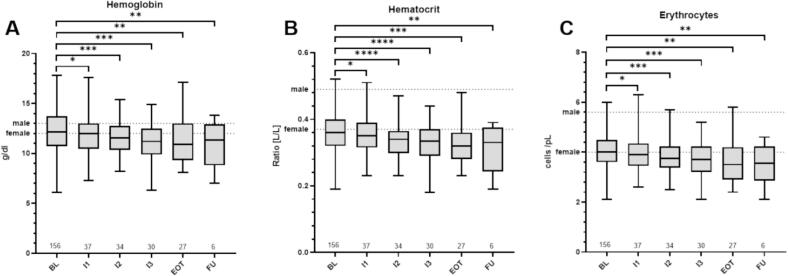
Overview over blood values for hemoglobin (A), hematocrit (B), and erythrocyte counts (C). Dotted lines indication individual thresholds for normal values. ns = p > 0.05, *=p ≤ 0.05, **=p ≤ 0.01, ***=p ≤ 0.001, ****= p ≤ 0.0001. BL = baseline, FU = follow up, RT = radiotherapy, EOT = End of Radiotherapy,

At baseline 84 patients (53.8 %) showed anemia. This increased up to 74.1 % of patients at the end of treatment (Fig. 1 **A**). At baseline 56.3 % of male and 50.0 % of female patient were anemic, while at EOT these numbers increased to 68.2 % of male and 100 % of female patients. Anemia was most prevalent in patients with metastatic disease, with 91.3 % (BL) and 100 % (EOT), respectively. Of note, in female patients the mean values for HB were below the lower limit of normal values all time points. For male patients mean HB values were above the lower threshold of normal range at BL, however fell below this threshold at I3 and continued this low until FU (Suppl. Tbl. 1). Red Blood cell counts, including hemoglobin levels (HB), hematocrit (Hct) and erythrocytes (Ery), significantly decreased over the course of treatment (BL vs EOT HB: p = 0.0011; Hct: p = 0.0007; Ery: p = 0.0011; BL vs FU HB: p = 0.0066; Hct: p = 0.0079; Ery: p = 0.0031) (Fig. 2).

ID was observed in 44.2 % of patients already before treatment start. Highest rates showed patients with metastatic disease (56.5 %) and patients with head and neck cancer (46.6 %), respectively (Fig. 1B). Surprisingly, at EOT only 29.6 % out of 27 patients showed ID (Fig. 1B).

### Development of the iron balance during the course of irradiation

**Fig. 3:**
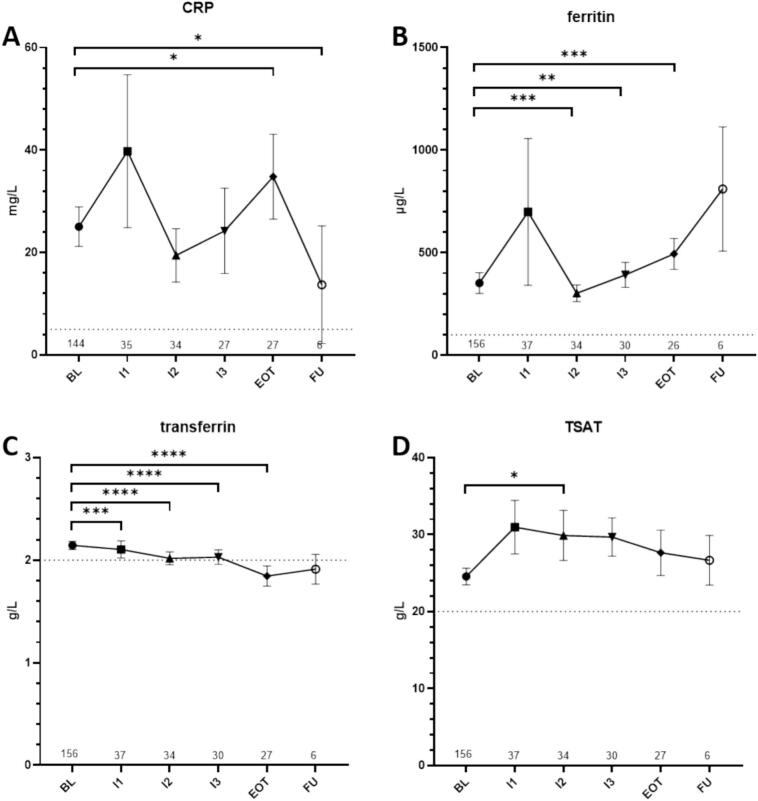
Overview over blood values for CRP (A), ferritin (B), transferrin(C), and TSAT (D). Shown are means and standard-errors of the mean. Dotted lines indication individual thresholds for normal values. ns = p > 0.05, *=p ≤ 0.05, **=p ≤ 0.01, ***=p ≤ 0.001, ****= p ≤ 0.0001. BL = baseline, CRP= C-reactive protein, FU = follow up, RT = radiotherapy, EOT = End of Radiotherapy.

As expected, systemic inflammation measured by CRP significantly increased during the course of irradiation with a mean at BL of 25.0 mg/L and 34.8 mg/ L at EOT (p = 0.024) (Fig. 3A). After completion of radiotherapy CRP levels dropped to 13.7 mg/L. Similar results were obtained for the acute phase protein ferritin with continuous increase during treatment (mean: BL = 351.9 µg/L; EOT = 494.2 µg/L; P = 0.0009) (Fig. 2B). Mean levels of transferrin decreased over time and were below the lower threshold (normal range: 2.0–3.6 g/L) at EOT (BL = 2.14 g/L; EOT = 1.84 g/L; p < 0.0001) (Fig. 2C). Over all patients, level of TSAT (Fig. 2D) did not show significant changes comparing baseline to end of treatment values (p = 0.086). A chi-squared test was used to compare ID with different other parameters including systemic therapy, cancer type, gender, and alcohol or nicotine abuse. Results showed a significant association between low TSAT at baseline and systemic therapy application (x^2(1)^ = 6.653, p = 0.01, ϕ = 0.208), chemotherapy (x^2(3)^ = 10.893, p = 0.012, ϕ = 0.266) and immunotherapy (x^2(3)^ = 11.579, p = 0.009, ϕ = 0.274). Low TSAT at EOT was significantly associated with alcohol abuse (x^2(2)^ = 6.356, p = 0.042, ϕ = 0.515) (Suppl. Tbl. 2, 3A).

Furthermore, baseline TSAT correlated with CRP, iron, ferritin, HB, Hct, leucocyte, and thrombocyte levels (Suppl. Tbl. 3B). TSAT at end of treatment negatively correlated with NLR and PLR (r = -0.46, p = 0.019; r = -0.5, p = 0.011) as well as leucocytes and thrombocyte counts (r = -0.401, p = 0.038; r = -0.456, p = 0.017). Associations were also found for iron and ferritin levels as expected (r = 0.789, p < 0.001; r = 0.468, p = 0.016).

### Differential effect of radiotherapy on transferrin saturation in patients with repeated measurements

**Fig. 4:**
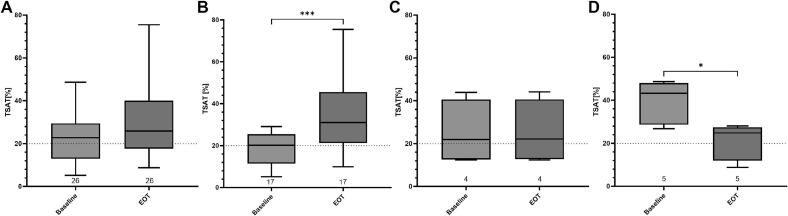
Overview over values for transferrin saturation at Baseline and EOT for matching samples over all patients (A), and patients with increase (B), decrease (D) and no changes (C) between baseline and end of treatment. Dotted line indicating normal threshold at a TSAT of 20 %. Shown are median and 25 and 75 quartiles. ns = p > 0.05, *=p ≤ 0.05, **=p ≤ 0.01, ***=p ≤ 0.001, ****= p ≤ 0.0001. BL = baseline, FU = follow up, RT = radiotherapy, EOT = End of Radiotherapy,

A subgroup analysis for TSAT of patients with repeated measurements at the beginning and end of treatment also showed no changes over the course of radiotherapy when considering the entire patient population (BL vs. EOT (mean (SD)): 23.7 (12.2) vs. 29.1 (15.5); p = 0.113). However, we were able to identify patients with three distinct divergent dynamics that showed (i) significant increase (n = 17, p = 0.0006), (ii) no changes (n = 4, p > 0.506), and (iii) significant decrease (n = 5, p = 0.001) of TSAT (Fig. 4**, Suppl. Fig.2**). As a next step we evaluated, which patients used a feeding tube as feeding tube nutrition contains iron. The German Nutrition Society (DGN) recommends a daily iron intake of 11 mg for adult men and postmenopausal women. Premenopausal women should consume 14–16 mg of iron per day to cover their requirements [9]. Standard feeding tube nutrition contains between 1.1 mg/100 ml and 1.6 mg/100 ml iron, depending on the company. For a standard intake of 1500 kcal this equals an intake of 11–24 mg iron per daily, therefore fulfilling aforementioned recommendations. Of note, oral iron substitution via medication for iron deficiency often includes treatment with 100–300 mg iron per day. However, irrespectively of oral iron substitution, 4 out of 5 patients with decreased TSAT used a feeding tube during radiotherapy treatment. Patients showing no change to their TSAT did not use feeding tubes regularly. Of note, 8 out of 17 patients (47.1 %) showing increase of TSAT used a feeding tube. All of these patients suffered from Head and Neck cancer. Patient characteristics of patients with matching samples are summarized in supplementary Table 4. Interestingly, patients with decrease of TSAT during radiotherapy were disproportionately frequently smokers and suffered from active alcohol abuse. Also 80 % of these patients were treated for HNSCC. Of note, patients with no change in TSAT had smallest PTV and GTV Volumes.

## Discussion

There are three key findings from this study: Our study confirmed the exceptionally high prevalence of anaemia of 53.8 % in patients with malignancies before undergoing radiotherapy and this prevalence is further increased to 74.1 % in the course of radiotherapy. Furthermore, 44.2 % of patients showed ID at baseline. Surprisingly, this number dropped to 29.6 % at EOT. Interestingly, we observed that oral supplementation of iron via feeding tube nutrition did not appear to affect the progression of ID from baseline to end of treatment.

Our results on prevalence of anemia are in line with a previous prospective cohort study done by Ludwig et al. [1]. Although their patient cohort included patients with other tumor entities, e.g. 21.7 % patients with breast cancer and only 5 % of patients with Head and neck cancer, Ludwig et al also reported anemia in 71.8 % of patients receiving combinational treatment with both radiation and chemotherapy. In our study, we expanded previous knowledge for the investigation of ID in these patients. ID also showed high prevalence at baseline measurements. ID at baseline was associated with systemic therapy, which is most probably due to 16.5 % of patients with systemic treatment receiving neoadjuvant chemo- or immunotherapy. An unexpected finding in our study was however, that prevalence of ID declined in the course of the treatment. This decline is most probably associated with an increase of hematotoxicity of systemic therapy as a cause for anemia as 66 % of patients received systemic therapy (85 % of these with concurrent chemotherapy). Interestingly, low TSAT at end of treatment was associated with alcohol abuse in the clinical history of the patient. This suggests that impaired liver function due to current or former alcohol abuse could contribute to an impaired hepatic protein synthesis of transferrin in the liver and thus result in decline of TSAT [10].

Investigation of development of TSAT in the course of the RCTx revealed differential results with some patients showing increase, no effect or decrease. However, the trajectory of TSAT levels was not affected by oral iron substitution via feeding tube nutrition as part of supplemental nutritional strategies. This finding underlines that oral substitution of iron in patients with cancer may not be successful to treat iron-deficient anemia in all patients undergoing radiotherapy. Oral uptake of iron is known to be merely 10–15 % in normal physiologic conditions and is even further reduced in patients with chronic inflammation such as patients with cancer. This restriction is related to the upregulation of hepcidin, the main regulating hormone of iron metabolism, mainly via IL-6 and IL1β [11], [12]. Hepcidin in turn downregulates the iron exporter ferroportin (FPN1) [13], resulting in decreased gastrointestinal iron resorption [14]. Hence, intravenous iron treatment may be more effective to improve iron status in these patients.

Comparison of oral versus intravenous iron substitution in a cohort of patients with gynecologic cancer undergoing chemotherapy showed higher mean hemoglobin and hematocrit levels after i.v. treatment [15]. Similar results have been obtained for patients suffering from chronic heart failure and only i.v. iron substitution is recommended by heart failure guidelines [16], [17]. In a study on 75 patients treated with platinum-based RCTx significantly less patients required blood transfusion when treated with intravenous iron sucrose infusion compared to control (40 vs 64 %) [18]. Similarly, HB levels of patients treated with intravenous and oral iron rose during and after RT, while patients undergoing transfusions in combination with oral iron showed decline [19].

Diagnosis and treatment of anemia and especially iron-deficient anemia in patients with cancer and thus chronic inflammation remains challenging [6]. Prevalence of ID in patients with solid tumors is high and has been shown to be associated with poor performance status [8]. However, definition of the diagnosis of ID varies between different medical societies and available evidence in patients with cancer remains limited, especially for patients undergoing RCTx [20]. The challenge of diagnosing ID in patients with cancer has also been addressed in the practice guidelines for the laboratory diagnosis of FID [21].

Our study had some limitations: The cohort of patients studied represents a heterogeneous group in terms of tumor entity, tumor size and individual risk factors. Also, the limited sample size, especially for repeated measurements, and the retrospective nature of the analysis must be considered when interpreting the results of this study. Therefore, the significance of subgroup analysis, for example concerning the prevalence of ID in patients with different cancer entities or the impact of treatment regimens including the use of chemotherapy on prevalence or induction of ID, remains limited. Larger prospective cohort studies should be performed to validate the findings of this study. In particular, data on consumption quantity, concrete time period of use, and individual feeding tube nutrition (e.g. manufacturer) could not be recovered from patient records. For this reason, the data on the impact of oral iron supplementation via feeding tube nutrition on progression of ID should be understood as hypothesis-generating rather than confirmatory. For patients with current or former alcohol abuse, other etiologies of anemia, including vitamin B12 and folate deficiencies or impaired renal function, may play a significant role in the development of anemia prior and during RCTx, and could potentially amplify the effects of ID in these patient cohort. Future studies should include the evaluation of other causes of anemia to avoid this possible bias.

In conclusion, our study underscores the high prevalence of anemia in patients treated with radiotherapy and identified iron deficiency as a main cause. Our data highlight, that oral iron substitution may not be sufficient to treat ID in patients undergoing radio(chemo-)therapy. These results underline the need to improve our understanding of iron balance and how to best treat deficiencies in these patients. Future studies should focus on the impact of iron deficiency and anemia on HRQoL during radiotherapy and how to enhance patient outcomes through targeted iron supplementation strategies.

## Statements and declarations

Study conception and design were mainly provided by Franziska Hausmann, Felix Mehrhof and Wolfram Doehner. Material preparation, data collection and analysis were performed by Sophie Schumacher, Falk Fabian, and Paula Bergengruen. The first draft of the manuscript was written by Franziska Hausmann and all authors commented on previous versions of the manuscript. All authors read and approved the final manuscript.

The authors did not receive support from any organization for the submitted work. Franziska Hausmann and Felix Mehrhof are involved in a trial sponsored by Varian Medical Systems outside of this work. Franziska Hausmann reports research funding from the German Ministry of Education and Research and German Cancer Aid. Wolfram Döhner received personal fees from Aimediq, Bayer, Boeringer Ingelheim, Boston Scientific, Medtronic, Vifor Pharma, travel support from Pharmacosmos, and research support to the Institute from EU (Horizon 2020), German Ministry of Education and Research, German Center for Cardiovascular Research, Boehringer Ingelheim, Vifor Pharma. Daniel Zips reports that radiation oncology Charité receives technical support and research grants from Varian Medical Systems/Siemens Healthineers, Accuray, TheraPanacea and Sennewald. All other authors disclose no competing interest concerning this work.

This study was performed in line with the principles of the Declaration of Helsinki. Approval was granted by the Ethics Committee of Charité University Medicine Berlin (EA1/101/23).

## CRediT authorship contribution statement

**Sophie Schumacher:** Investigation, Writing – original draft, Writing – review & editing. **Falk Fabian:** Investigation, Writing – original draft, Writing – review & editing. **Paula Lehmann:** Writing – review & editing. **Daniel Zips:** Writing – review & editing. **Felix Mehrhof:** Methodology, Writing – review & editing. **Wolfram Doehner:** Conceptualization, Methodology, Supervision, Writing – review & editing. **Franziska Hausmann:** Conceptualization, Methodology, Formal analysis, Visualization, Writing – original draft, Writing – review & editing.

## Declaration of competing interest

The authors declare that they have no known competing financial interests or personal relationships that could have appeared to influence the work reported in this paper.
