# Supplementary material for: Iron deficiency in patients undergoing radiotherapy: Prevalence and clinical characteristics
Source: Clin Transl Radiat Oncol. 2026 Jan 18;57:101111. doi: 10.1016/j.ctro.2026.101111 (PMC12861250; doi:10.1016/j.ctro.2026.101111)
Supplement: Supplementary Data 1 [file mmc1.pptx]

## Slide 1
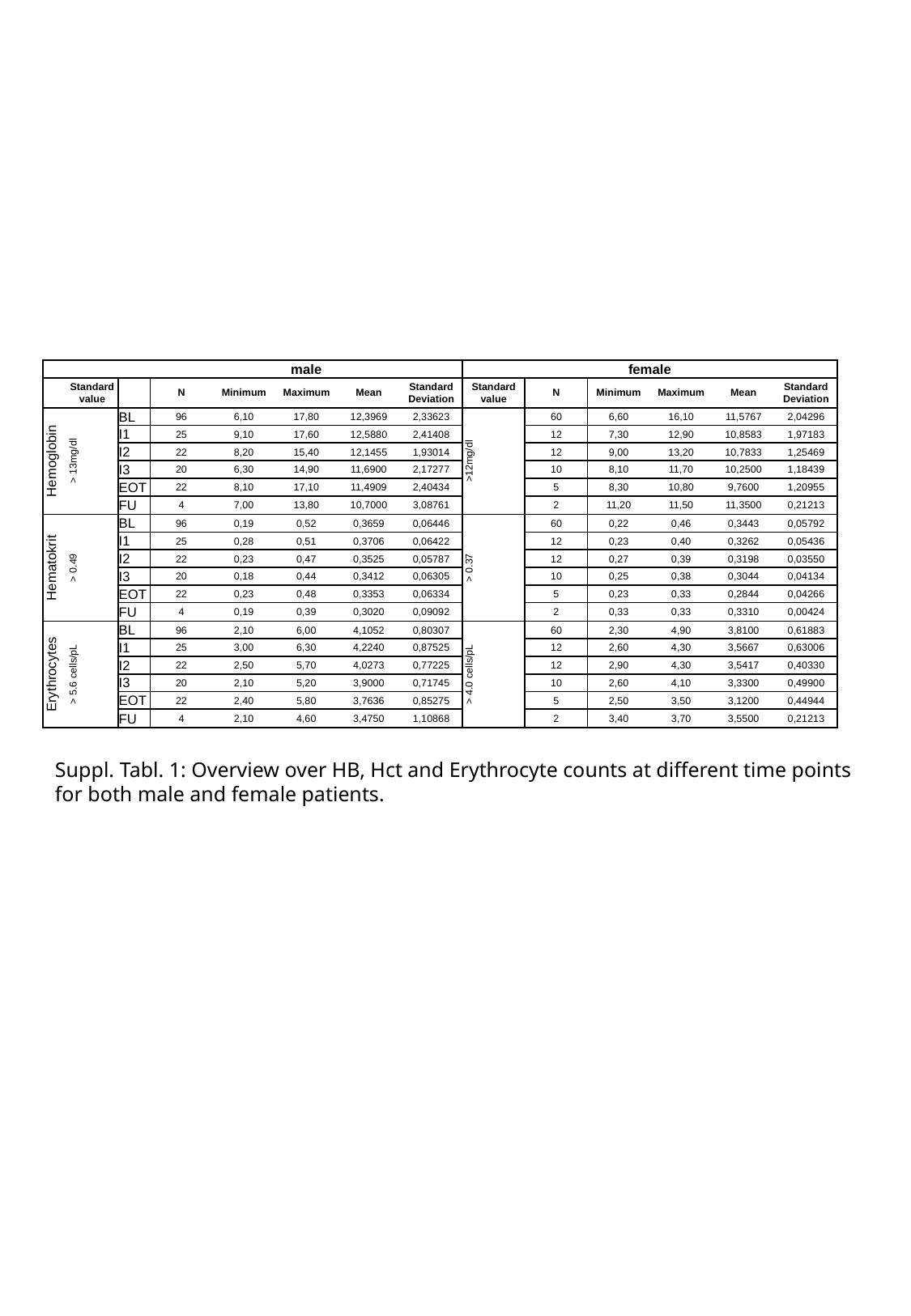

| | | | male | | | | | female | | | | | |
| --- | --- | --- | --- | --- | --- | --- | --- | --- | --- | --- | --- | --- | --- |
| | Standard value | | N | Minimum | Maximum | Mean | Standard Deviation | Standard value | N | Minimum | Maximum | Mean | Standard Deviation |
| Hemoglobin | > 13mg/dl | BL | 96 | 6,10 | 17,80 | 12,3969 | 2,33623 | >12mg/dl | 60 | 6,60 | 16,10 | 11,5767 | 2,04296 |
| | | I1 | 25 | 9,10 | 17,60 | 12,5880 | 2,41408 | | 12 | 7,30 | 12,90 | 10,8583 | 1,97183 |
| | | I2 | 22 | 8,20 | 15,40 | 12,1455 | 1,93014 | | 12 | 9,00 | 13,20 | 10,7833 | 1,25469 |
| | | I3 | 20 | 6,30 | 14,90 | 11,6900 | 2,17277 | | 10 | 8,10 | 11,70 | 10,2500 | 1,18439 |
| | | EOT | 22 | 8,10 | 17,10 | 11,4909 | 2,40434 | | 5 | 8,30 | 10,80 | 9,7600 | 1,20955 |
| | | FU | 4 | 7,00 | 13,80 | 10,7000 | 3,08761 | | 2 | 11,20 | 11,50 | 11,3500 | 0,21213 |
| Hematokrit | > 0.49 | BL | 96 | 0,19 | 0,52 | 0,3659 | 0,06446 | > 0.37 | 60 | 0,22 | 0,46 | 0,3443 | 0,05792 |
| | | I1 | 25 | 0,28 | 0,51 | 0,3706 | 0,06422 | | 12 | 0,23 | 0,40 | 0,3262 | 0,05436 |
| | | I2 | 22 | 0,23 | 0,47 | 0,3525 | 0,05787 | | 12 | 0,27 | 0,39 | 0,3198 | 0,03550 |
| | | I3 | 20 | 0,18 | 0,44 | 0,3412 | 0,06305 | | 10 | 0,25 | 0,38 | 0,3044 | 0,04134 |
| | | EOT | 22 | 0,23 | 0,48 | 0,3353 | 0,06334 | | 5 | 0,23 | 0,33 | 0,2844 | 0,04266 |
| | | FU | 4 | 0,19 | 0,39 | 0,3020 | 0,09092 | | 2 | 0,33 | 0,33 | 0,3310 | 0,00424 |
| Erythrocytes | > 5.6 cells/pL | BL | 96 | 2,10 | 6,00 | 4,1052 | 0,80307 | > 4.0 cells/pL | 60 | 2,30 | 4,90 | 3,8100 | 0,61883 |
| | | I1 | 25 | 3,00 | 6,30 | 4,2240 | 0,87525 | | 12 | 2,60 | 4,30 | 3,5667 | 0,63006 |
| | | I2 | 22 | 2,50 | 5,70 | 4,0273 | 0,77225 | | 12 | 2,90 | 4,30 | 3,5417 | 0,40330 |
| | | I3 | 20 | 2,10 | 5,20 | 3,9000 | 0,71745 | | 10 | 2,60 | 4,10 | 3,3300 | 0,49900 |
| | | EOT | 22 | 2,40 | 5,80 | 3,7636 | 0,85275 | | 5 | 2,50 | 3,50 | 3,1200 | 0,44944 |
| | | FU | 4 | 2,10 | 4,60 | 3,4750 | 1,10868 | | 2 | 3,40 | 3,70 | 3,5500 | 0,21213 |
Suppl. Tabl. 1: Overview over HB, Hct and Erythrocyte counts at different time points for both male and female patients.

## Slide 2
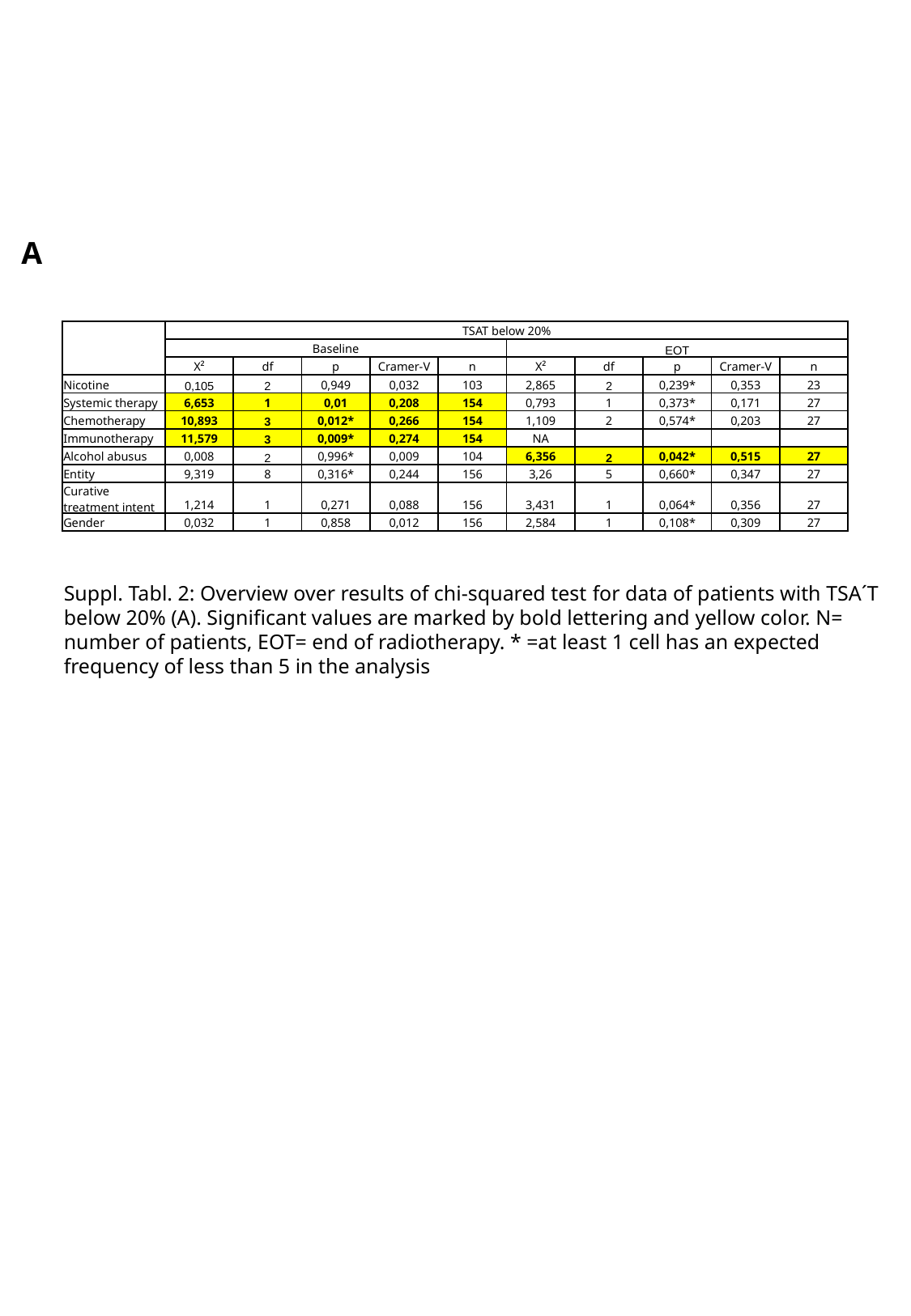

A
| | TSAT below 20% | | | | | | | | | |
| --- | --- | --- | --- | --- | --- | --- | --- | --- | --- | --- |
| | Baseline | | | | | EOT | | | | |
| | X² | df | p | Cramer-V | n | X² | df | p | Cramer-V | n |
| Nicotine | 0,105 | 2 | 0,949 | 0,032 | 103 | 2,865 | 2 | 0,239\* | 0,353 | 23 |
| Systemic therapy | 6,653 | 1 | 0,01 | 0,208 | 154 | 0,793 | 1 | 0,373\* | 0,171 | 27 |
| Chemotherapy | 10,893 | 3 | 0,012\* | 0,266 | 154 | 1,109 | 2 | 0,574\* | 0,203 | 27 |
| Immunotherapy | 11,579 | 3 | 0,009\* | 0,274 | 154 | NA | | | | |
| Alcohol abusus | 0,008 | 2 | 0,996\* | 0,009 | 104 | 6,356 | 2 | 0,042\* | 0,515 | 27 |
| Entity | 9,319 | 8 | 0,316\* | 0,244 | 156 | 3,26 | 5 | 0,660\* | 0,347 | 27 |
| Curative treatment intent | 1,214 | 1 | 0,271 | 0,088 | 156 | 3,431 | 1 | 0,064\* | 0,356 | 27 |
| Gender | 0,032 | 1 | 0,858 | 0,012 | 156 | 2,584 | 1 | 0,108\* | 0,309 | 27 |
Suppl. Tabl. 2: Overview over results of chi-squared test for data of patients with TSA´T below 20% (A). Significant values are marked by bold lettering and yellow color. N= number of patients, EOT= end of radiotherapy. * =at least 1 cell has an expected frequency of less than 5 in the analysis

## Slide 3
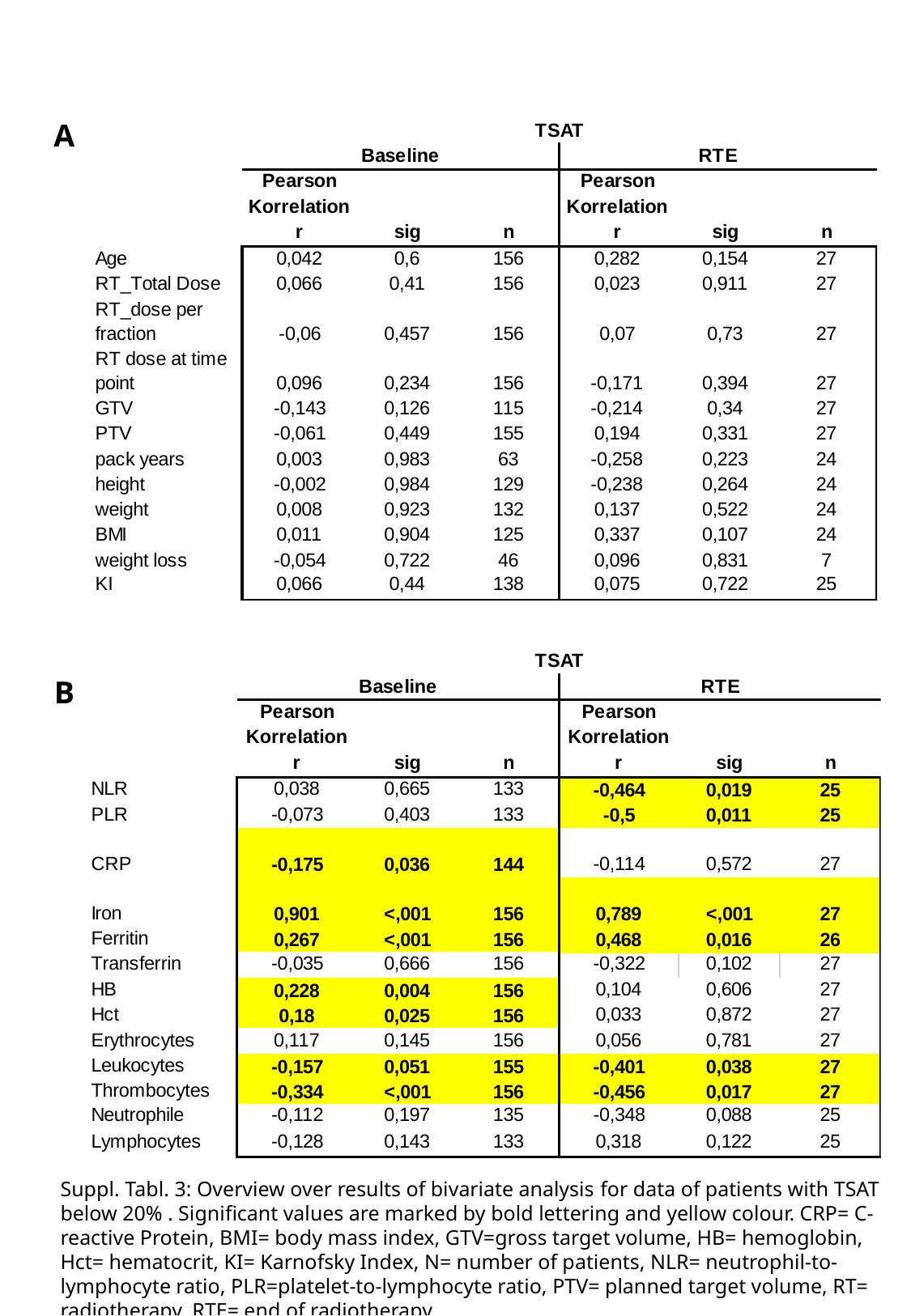

A
B
Suppl. Tabl. 3: Overview over results of bivariate analysis for data of patients with TSAT below 20% . Significant values are marked by bold lettering and yellow colour. CRP= C-reactive Protein, BMI= body mass index, GTV=gross target volume, HB= hemoglobin, Hct= hematocrit, KI= Karnofsky Index, N= number of patients, NLR= neutrophil-to-lymphocyte ratio, PLR=platelet-to-lymphocyte ratio, PTV= planned target volume, RT= radiotherapy, RTE= end of radiotherapy.

## Slide 4
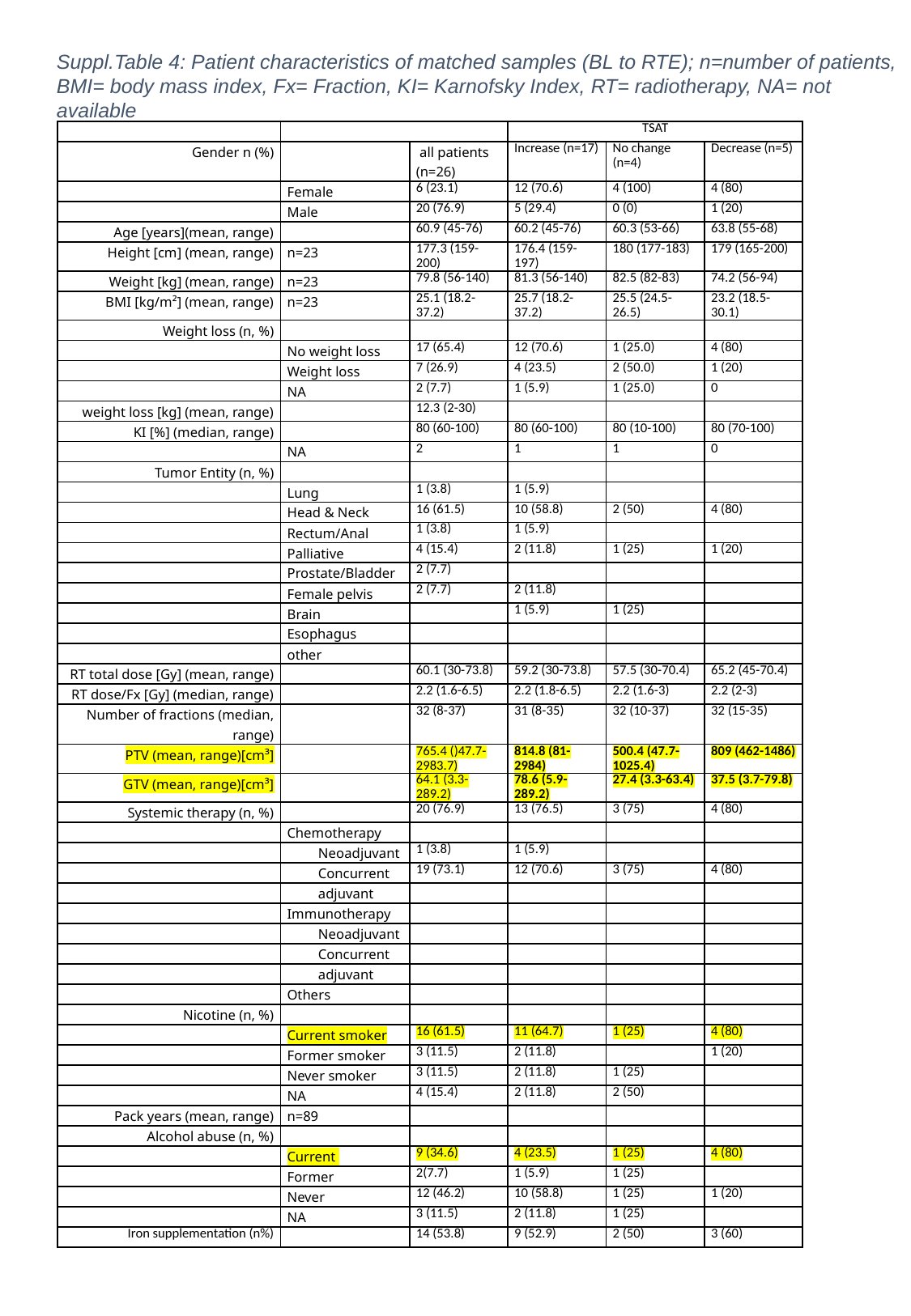

Suppl.Table 4: Patient characteristics of matched samples (BL to RTE); n=number of patients, BMI= body mass index, Fx= Fraction, KI= Karnofsky Index, RT= radiotherapy, NA= not available
| | | | TSAT | | |
| --- | --- | --- | --- | --- | --- |
| Gender n (%) | | all patients (n=26) | Increase (n=17) | No change (n=4) | Decrease (n=5) |
| | Female | 6 (23.1) | 12 (70.6) | 4 (100) | 4 (80) |
| | Male | 20 (76.9) | 5 (29.4) | 0 (0) | 1 (20) |
| Age [years](mean, range) | | 60.9 (45-76) | 60.2 (45-76) | 60.3 (53-66) | 63.8 (55-68) |
| Height [cm] (mean, range) | n=23 | 177.3 (159-200) | 176.4 (159-197) | 180 (177-183) | 179 (165-200) |
| Weight [kg] (mean, range) | n=23 | 79.8 (56-140) | 81.3 (56-140) | 82.5 (82-83) | 74.2 (56-94) |
| BMI [kg/m²] (mean, range) | n=23 | 25.1 (18.2-37.2) | 25.7 (18.2-37.2) | 25.5 (24.5-26.5) | 23.2 (18.5-30.1) |
| Weight loss (n, %) | | | | | |
| | No weight loss | 17 (65.4) | 12 (70.6) | 1 (25.0) | 4 (80) |
| | Weight loss | 7 (26.9) | 4 (23.5) | 2 (50.0) | 1 (20) |
| | NA | 2 (7.7) | 1 (5.9) | 1 (25.0) | 0 |
| weight loss [kg] (mean, range) | | 12.3 (2-30) | | | |
| KI [%] (median, range) | | 80 (60-100) | 80 (60-100) | 80 (10-100) | 80 (70-100) |
| | NA | 2 | 1 | 1 | 0 |
| Tumor Entity (n, %) | | | | | |
| | Lung | 1 (3.8) | 1 (5.9) | | |
| | Head & Neck | 16 (61.5) | 10 (58.8) | 2 (50) | 4 (80) |
| | Rectum/Anal | 1 (3.8) | 1 (5.9) | | |
| | Palliative | 4 (15.4) | 2 (11.8) | 1 (25) | 1 (20) |
| | Prostate/Bladder | 2 (7.7) | | | |
| | Female pelvis | 2 (7.7) | 2 (11.8) | | |
| | Brain | | 1 (5.9) | 1 (25) | |
| | Esophagus | | | | |
| | other | | | | |
| RT total dose [Gy] (mean, range) | | 60.1 (30-73.8) | 59.2 (30-73.8) | 57.5 (30-70.4) | 65.2 (45-70.4) |
| RT dose/Fx [Gy] (median, range) | | 2.2 (1.6-6.5) | 2.2 (1.8-6.5) | 2.2 (1.6-3) | 2.2 (2-3) |
| Number of fractions (median, range) | | 32 (8-37) | 31 (8-35) | 32 (10-37) | 32 (15-35) |
| PTV (mean, range)[cm³] | | 765.4 ()47.7-2983.7) | 814.8 (81-2984) | 500.4 (47.7-1025.4) | 809 (462-1486) |
| GTV (mean, range)[cm³] | | 64.1 (3.3-289.2) | 78.6 (5.9-289.2) | 27.4 (3.3-63.4) | 37.5 (3.7-79.8) |
| Systemic therapy (n, %) | | 20 (76.9) | 13 (76.5) | 3 (75) | 4 (80) |
| | Chemotherapy | | | | |
| | Neoadjuvant | 1 (3.8) | 1 (5.9) | | |
| | Concurrent | 19 (73.1) | 12 (70.6) | 3 (75) | 4 (80) |
| | adjuvant | | | | |
| | Immunotherapy | | | | |
| | Neoadjuvant | | | | |
| | Concurrent | | | | |
| | adjuvant | | | | |
| | Others | | | | |
| Nicotine (n, %) | | | | | |
| | Current smoker | 16 (61.5) | 11 (64.7) | 1 (25) | 4 (80) |
| | Former smoker | 3 (11.5) | 2 (11.8) | | 1 (20) |
| | Never smoker | 3 (11.5) | 2 (11.8) | 1 (25) | |
| | NA | 4 (15.4) | 2 (11.8) | 2 (50) | |
| Pack years (mean, range) | n=89 | | | | |
| Alcohol abuse (n, %) | | | | | |
| | Current | 9 (34.6) | 4 (23.5) | 1 (25) | 4 (80) |
| | Former | 2(7.7) | 1 (5.9) | 1 (25) | |
| | Never | 12 (46.2) | 10 (58.8) | 1 (25) | 1 (20) |
| | NA | 3 (11.5) | 2 (11.8) | 1 (25) | |
| Iron supplementation (n%) | | 14 (53.8) | 9 (52.9) | 2 (50) | 3 (60) |

## Slide 5
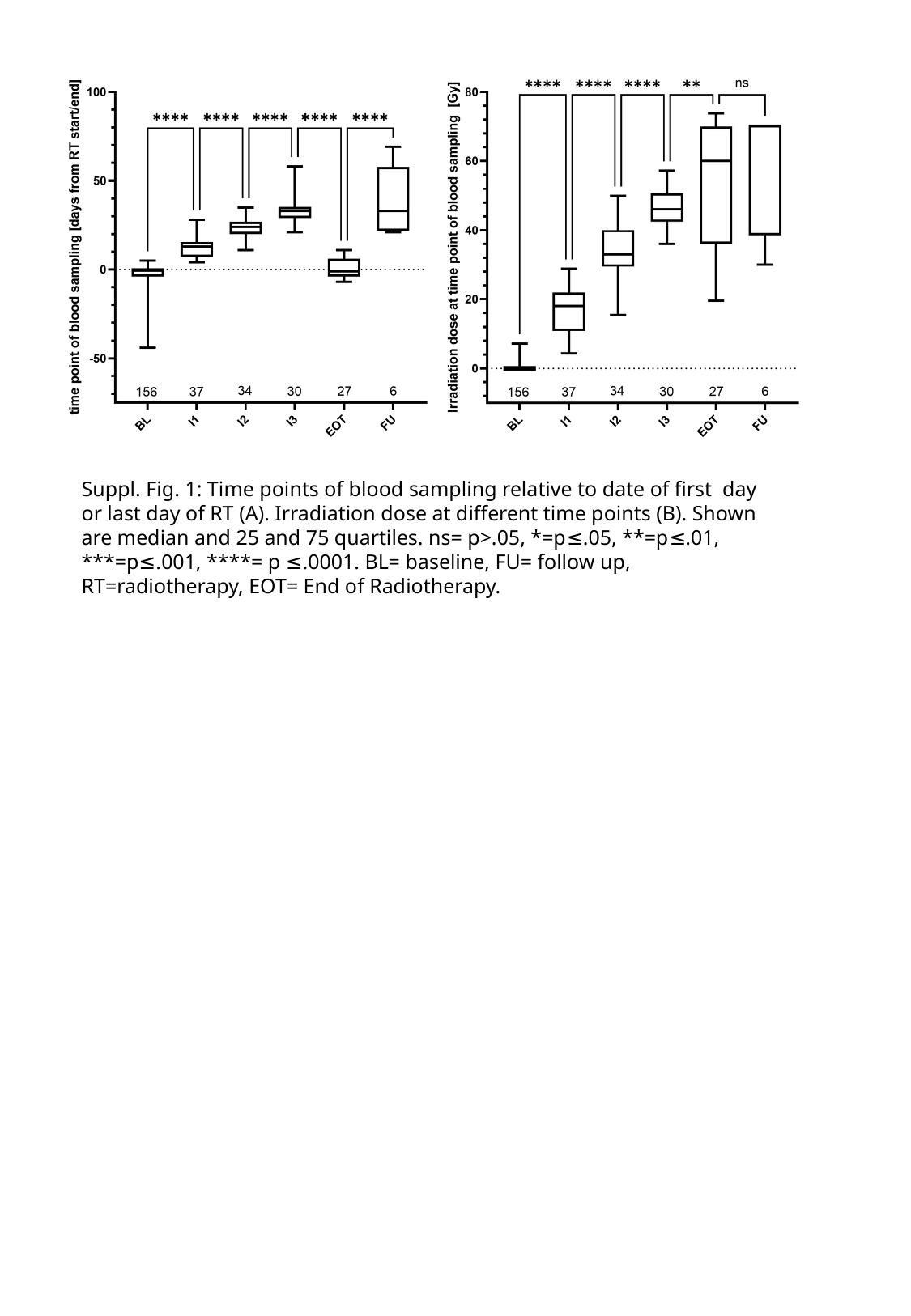

Suppl. Fig. 1: Time points of blood sampling relative to date of first day or last day of RT (A). Irradiation dose at different time points (B). Shown are median and 25 and 75 quartiles. ns= p>.05, *=p≤.05, **=p≤.01, ***=p≤.001, ****= p ≤.0001. BL= baseline, FU= follow up, RT=radiotherapy, EOT= End of Radiotherapy.

## Slide 6
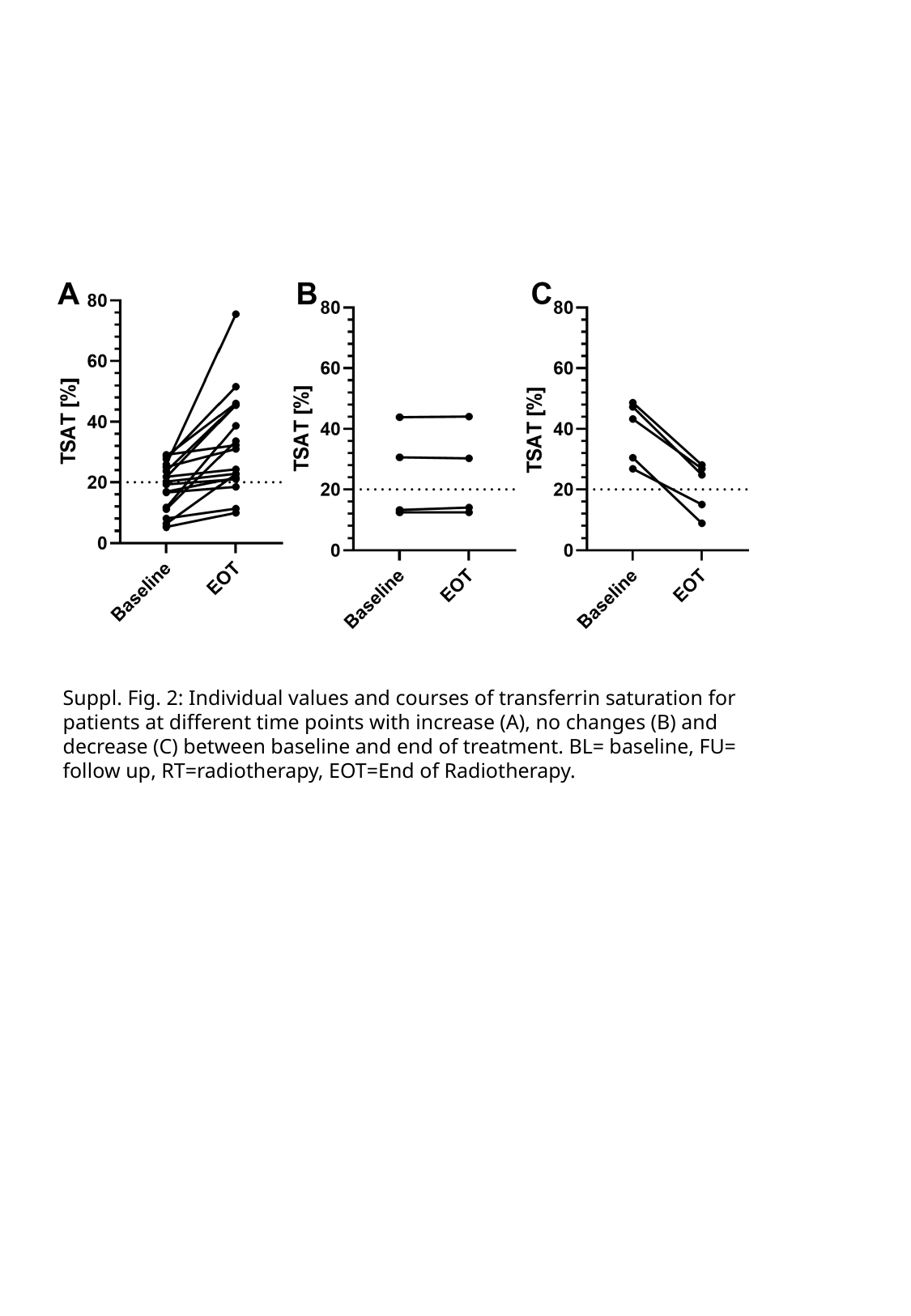

Suppl. Fig. 2: Individual values and courses of transferrin saturation for patients at different time points with increase (A), no changes (B) and decrease (C) between baseline and end of treatment. BL= baseline, FU= follow up, RT=radiotherapy, EOT=End of Radiotherapy.
